# Anxiolytic and Antidepressant‐Like Effects of a Probiotic Mixture HMLH123 Potentially via the Serotonergic Pathway in Wistar Rats

**DOI:** 10.1155/bn/6680245

**Published:** 2026-03-23

**Authors:** Gilberto Uriel Rosas-Sánchez, León Jesús Germán-Ponciano, Angélica Yanet Nápoles-Medina, Blanca Rosa Aguilar-Uscanga, Mario Eduardo Flores-Soto, Cesar Soria-Fregozo

**Affiliations:** ^1^ Programa de Estancias Posdoctorales por Mexico, SECIHTI, Centro Universitario de Los Lagos, Universidad de Guadalajara, Lagos de Moreno, Jalisco, Mexico, udg.mx; ^2^ Laboratorio de Neurofarmacología, Instituto de Neuroetología, Universidad Veracruzana, Xalapa, Veracruz, Mexico, uv.mx; ^3^ Laboratorio de Neurobiología Celular y Molecular, División de Neurociencias, Centro de Investigación Biomédica de Occidente (CIBO) Instituto Mexicano del Seguro Social, Guadalajara, Jalisco, Mexico; ^4^ Departamento de Farmacobiología, Laboratorio de Microbiología Industrial, Centro Universitario de Ciencias Exactas e Ingenierías, Universidad de Guadalajara, Guadalajara, Jalisco, Mexico, udg.mx; ^5^ Departamento de Ciencias de la Tierra y de la Vida, Centro Universitario de Los Lagos, Universidad de Guadalajara, Lagos de Moreno, Jalisco, Mexico, udg.mx

**Keywords:** antidepressant, anxiolytic, elevated plus maze, forced swim test, *Lactiplantibacillus* sp., microbiota–gut–brain axis, open field test

## Abstract

Anxiety and depression are a major health problem worldwide with increasing prevalence and limitations in pharmacologic treatment. The gut–brain axis has emerged as a potential therapeutic target, with probiotics showing promise in the regulation of mental health. To evaluate the anxiolytic and antidepressant‐like effects of a probiotic mixture HMLH123 and its pharmacologic interaction with fluoxetine, 60 male Wistar rats were divided into six experimental groups that received either vehicle, fluoxetine (0.5 or 1 mg/kg), probiotic mixture HMLH123 200 *μ*L to a concentration of 10^9^ CFU/mL (*Lactiplantibacillus* sp. LH01, *Lactiplantibacillus* sp. LH02, and *Lactiplantibacillus* sp. LH3), or a combination of fluoxetine and probiotic mixture HMLH123. Behavioral assessments included the locomotor activity test (LAT), the elevated plus maze (EPM), and the forced swim test (FST). Data were analyzed using one‐way ANOVA with a significance level of *p* ≤ 0.05. Administration of probiotic mixture HMLH123 or in combination with fluoxetine significantly increased the time spent in the open arms of the EPM, indicating anxiolytic‐like effects. In the FST, groups treated with probiotic mixture HMLH123 showed reduced immobility time and increased swimming behavior, suggesting a possible modulation of the serotonergic system. These findings support the potential of probiotic mixture HMLH123 as a preclinical alternative approach for the treatment of anxiety and depression. While no synergistic interaction with fluoxetine was observed, treatment with the probiotic mixture showed positive effects independently. Further research should explore its mechanisms of action and therapeutic applications as a standalone intervention.

## 1. Introduction

Anxiety and depression have become a major global health problem, with their prevalence increasing by up to 25% worldwide since the COVID‐19 pandemic [1]. Anxiety disorders currently affect over 374 million people worldwide [2], while depression impacts around 332 million people, with a prevalence of 5% in adults and 5.7% in those over 60 years [3]. These disorders often co‐occur, exacerbating symptoms, reducing quality of life, and increasing the risk of suicide. Despite their widespread efficacy, current pharmacological treatments have significant limitations, including low remission rates and adverse effects, highlighting the need for safer and more effective therapeutic strategies [4].

The pharmacological treatment of anxiety and depression is associated with some limitations. A considerable proportion of patients fails to achieve significant symptom relief, alongside side effects such as weight gain, drowsiness, and gastrointestinal problems [5], which can significantly affect patients′ quality of life. In addition, the delayed onset of the therapeutic effect, which often only becomes noticeable after weeks, leads to poor adherence to treatment, as many patients discontinue the medication early [6]. These challenges have led to a growing interest in complementary approaches, including interventions targeting the gut–brain axis.

New findings have shed light on the role of the microbiota–gut–brain (MGB) axis in the regulation of mental health. This bidirectional communication system between the gut and the brain has profound effects on mood regulation. Dysbiosis or imbalance in the gut microbiota has been implicated in the onset and progression of neuropsychiatric disorders, including anxiety and depression [7]. The gut microbiota influences the production of neurotransmitters, particularly serotonin, and modulates systemic inflammation, which is associated with mood disorders [8]. In addition, gut‐derived metabolites such as short‐chain fatty acids (SCFAs) have neuroprotective and anti‐inflammatory effects, contributing to mental well‐being [9]. These findings suggest that interventions that modulate the composition of the gut microbiota may offer novel therapeutic potential.

Probiotics, live microorganisms known for their health benefits, are increasingly recognized for their potential to modulate gut health and consequently mental health. Various probiotic strains, such as *Lactobacillus rhamnosus* JB‐1, have shown anxiolytic and antidepressant‐like effects in animal models by altering the concentration of neurotransmitters in the brain and reducing stress‐induced behaviors [10]. In addition, strains such as *Lactobacillus fermentum*, *Lactobacillus reuteri*, and *Lactobacillus plantarum* have shown anxiolytic and antidepressant properties in preclinical models, particularly in rodents. These effects have been associated with a reduction in inflammatory processes and downregulation of hypothalamic–pituitary–adrenal (HPA) axis activity [11]. Furthermore, their mechanism of action is associated with the activation of neurotrophic factors such as brain‐derived neurotrophic factor (BDNF) [12], modulation of the serotonergic neurotransmission pathway [13], and regulation of the gut microbiota and fecal metabolites [14].

Given the growing body of evidence supporting the role of probiotics in modulating the gut–brain axis and their potential therapeutic effect on anxiety and depression, further research is needed to explore their efficacy and potential interactions with conventional pharmacological treatments. Therefore, the aim of the present study was to investigate the anxiolytic and antidepressant‐like effects of a mixture of probiotics HMLH123 (*Lactiplantibacillus* sp. LH01, *Lactiplantibacillus sp*. LH02, and *Lactiplantibacillus sp*. LH3) and their pharmacological interaction with the antidepressant fluoxetine. By investigating the synergy between probiotics and conventional pharmacotherapy, this study is aimed at contributing to the neuropharmacology of affective disorders and providing new perspectives for microbiota‐based interventions for mental health.

## 2. Materials and Methods

### 2.1. Ethics Statements

All experimental procedures were conducted in accordance with the Guide for the Care and Use of Laboratory Animals published by the National Institutes of Health [15] and the Norma Oficial Mexicana para el Uso y Cuidado de Animales de Laboratorio [16]. Every effort was made to minimize animal discomfort and reduce the number of animals included, adhering to the replace, reduce, refine (3R) principles of preclinical research [17]. This study was reviewed and approved by the Local Health Research Ethics Committee of Centro Universitario de los Lagos, University of Guadalajara, under Institutional Registration Number CICUAL‐CULAGOS‐UDG: 2025_001.

### 2.2. Animals

Sixty male Wistar rats (2 months old, 250–300 g at the start of the experiments) were housed in Plexiglas cages, four rats per cage (44‐cm width × 33‐cm length × 20‐cm height), under a 12/12 h light/dark cycle (lights on at 07:00 h), with an average room temperature of 25°C (±1°C), and they had free access to purified water and food (Purina Pellets, Agribrands Purina Mexico, Mexico City, Mexico) throughout the study period. All experimental sessions were conducted between 09:00 and 12:00 h.

### 2.3. Probiotic Mixture HMLH123 Production and Drugs

*Lactiplantibacillus* sp. LH01, *Lactiplantibacillus sp.* LH02, and *Lactiplantibacillus sp.* LH3 were sourced from human milk and belong to the strain collection of the “Laboratorio de Investigación Leche humana” at the University of Guadalajara. The strains were cultured in de Man Rogosa Sharpe medium (Cat. 288130, Difco) supplemented with 10% Nutraflora (Ingredion), incubated at 37°C for 24 h, and harvested via centrifugation at 4500 rpm for 10 min; they were later washed with sterile physiological saline. The bacteria were suspended in sterile physiological saline and adjusted to a concentration of 10^9^ CFU/mL for oral administration of 200 *μ*L. The suspension was kept at freezing temperature (−20°C) until use [18]. The dose of fluoxetine (1 mg/kg) used in this study has been validated in previous studies for its antidepressant‐like effects in the forced swimming test [19]. This dose was initially proposed as the minimum effective dose capable of producing sustained antidepressant effects over time [20] and has subsequently been confirmed by other investigations demonstrating its efficacy in similar behavioral paradigms. Fluoxetine (Prozac) was sourced from Eli‐Lilly Company (Mexico City, Mexico). Administration was performed using a curved stainless‐steel oral gavage needle (18G × 3.000 with 2.5‐mm ball tip, Cadence Inc., Staunton, Virginia, United States), connected to a 1‐mL disposable syringe (Terumo Medical de México, Mexico City). This route was selected to resemble human oral drug intake, allowing for gastrointestinal processing that may affect drug metabolism and therapeutic response.

### 2.4. Experimental Groups

A randomized controlled experiment was conducted with six independent groups, each consisting of 10 rats. The groups included a control group that received the vehicle (purified water), fluoxetine 0.5 mg/kg, fluoxetine 1 mg/kg, probiotic mixture HMLH123, the interaction of fluoxetine 0.5 mg/kg and probiotic mixture HMLH123 (I‐1), and the interaction of fluoxetine 1 mg/kg and probiotic mixture HMLH123 (I‐2). Prior to any drug administration, all rats underwent a 5‐min pretest in LAT and a 15‐min pretest in FST. The results of the pretests were discarded from the statistical analysis. In the forced swimming pretest, rats were exposed to a stressful, aversive situation represented by swimming, which triggers the development of hopelessness behavior [21]. Twenty‐four hours later, the drug treatments were initiated, and their effect was evaluated on Day 28 of treatment, 1 h after the corresponding administration.

### 2.5. Behavioral Test

#### 2.5.1. Elevated Plus Maze

The apparatus was constructed from wood and placed in a room illuminated at 40 lux. The apparatus consisted of two opposing open and closed arms arranged in a plus configuration. The dimensions of the open arms were 50 × 10 cm (length × width), while the closed arms measured 50 × 10 × 40 cm (length × width × height). The entire maze was elevated 50 cm from the floor. Rats were individually placed at the center of the maze, facing an open arm [22]. The following variables were assessed: (i) time (in seconds) spent on the open arms, (ii) number of entries into the open arms, (iii) total number of arm entries (open arms + closed arms), and (iv) percentage of open arm entries ([open arm entries/total arm entries] × 100). Additionally, the anxiety index (AI) was calculated according to the following formula: AI = 1 − [([Open arm time/Test duration] 131 + [Open arm entries/Total number of entries])/2] [23].

#### 2.5.2. Locomotor Activity Test

The changes in spontaneous locomotor activity were evaluated by individually placing the rats into an opaque Plexiglas cage (44 × 33 cm, base; 20‐cm high) with the floor delineated into 12 squares (11 × 11 cm). Variables such as the number of crossings and time spent in seconds grooming and rearing were evaluated. Rats were considered to have crossed from one square to another (crossing) when the hind legs crossed the line from one square to another. Grooming included all self‐directed behaviors of cleaning from head, ears, limbs, and anal–genital region [24]. Rearing was measured when rats explored the cage in a vertical position standing on its rear limbs. Rearing was evaluated as being a behavior related to exploration [25]. After each rat was tested, the locomotor activity cage was carefully cleaned with a 10% alcohol solution to remove the scent of the previously evaluated rat. After the LAT, rats were subjected to the FST. Approximately 2 min elapsed between tests.

#### 2.5.3. Forced Swim Test

Rats were individually placed in a rectangular tank (50 × 30 × 60 cm) filled with 30 cm of water at 25°C ± 1°C, which has been validated for identifying substances with potential antidepressant‐like effects [26]. The following variables were evaluated: (i) latency to the first immobility (in seconds), defined as the time elapsed from the moment the rat was introduced into the water until the first episode of immobility; (ii) total time of immobility (in seconds), which was recorded when the rat remained floating for more than 2 s without vigorous movements; (iii) swimming behavior (in seconds), characterized by active movements of the front legs with directed horizontal actions, such as crossing between the quadrants of the tank and turning; and (iv) climbing behavior (in seconds), defined as upward movements of the front legs along the walls of the tank. All sessions were video recorded for 5 min using a Nikon D3300 camera with an 18–55‐mm lens, and behavioral variables were measured by two blinded observers with a concordance level of 95% using an *ex profeso* software for behavioral analysis.

### 2.6. Statistical Analysis

Sigma Plot 12 software was used to perform statistical analysis. Data were analyzed using one‐way analysis of variance (ANOVA) to assess the effect of treatments on behavior in the EPM, LAT, and FST, followed by the Student–Newman–Keuls post hoc test. The value of *p* ≤ 0.05 was considered statistically significant. The results are presented as the mean ± standard error of the mean (SEM).

## 3. Results

### 3.1. Elevated Plus Maze

Significant differences were observed in the time spent on the open arms by treatments (*F*_5,54_ = 2.820; *p* = 0.025). Post hoc analysis revealed significant differences between treatments in both the time spent on the open arms and the AI (*F*_5,54_ = 2.820; *p* = 0.025 for open arms; *F*_5,54_ = 3.611; *p* = 0.007 for AI). The time spent on the open arms was significantly longer (*p* < 0.05) in the 0.5 and 1 mg/kg fluoxetine, probiotic mixture HMLH123, I‐1, and I‐2 groups compared to the vehicle group (Figure 1a). Conversely, the AI was significantly lower (*p* < 0.05) in these same treatment groups compared to the vehicle group (Figure 1b).

Figure 1EPM performance. (a) Time spent in the open arms was significantly higher in the groups treated with Flx 0.5, Flx 1, Pro, I‐1, and I‐2 (*p* = 0.025) compared to the Veh group, (b) while the anxiety index was significantly lower in these treatment groups (*p* = 0.007 vs. Veh, one‐way ANOVA; Student–Newman–Keuls post hoc test). Abbreviations: Veh: vehicle; Flx 0.5: fluoxetine 0.5 mg/kg; Flx 1: fluoxetine 1 mg/kg; Pro: probiotics HMLH123; I‐1: fluoxetine (0.5 mg/kg) + probiotics HMLH123; I‐2: fluoxetine (1 mg/kg) + probiotics HMLH123.

**Figure:**
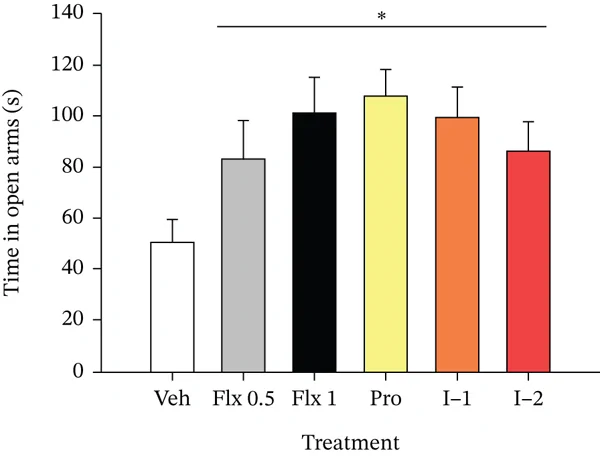
(a)

**Figure:**
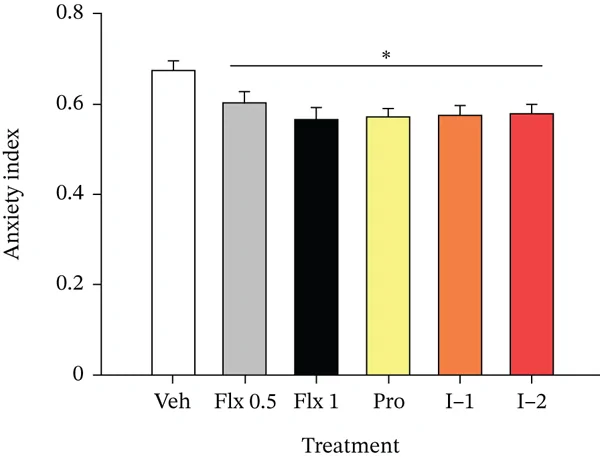
(b)

### 3.2. Locomotor Activity Test

The results of the LAT are summarized in Table 1. The data summarized in Table 1 shows similar mean values among groups in crossings in the LAT test (vehicle: 50.20 ± 3.83; fluoxetine 0.5 mg/kg: 48.80 ± 5.83; fluoxetine 1 mg/kg: 46.40 ± 7.71; HMLH123: 58.00 ± 7.15; I‐1: 66.20 ± 7.79; I‐2: 67.00 ± 10.26). Statistical comparisons revealed no significant differences among treatments (*F*_5,54_ = 1.501; *p* = 0.205, NS). Similarly, mean grooming time was comparable among groups (vehicle: 61.47 ± 11.28; fluoxetine 0.5 mg/kg: 55.52 ± 8.20; fluoxetine 1 mg/kg: 58.54 ± 13.29; HMLH123: 57.50 ± 12.77; I‐1: 65.94 ± 8.36; I‐2: 43.24 ± 9.01), and statistical analysis revealed no significant differences between treatments (*F*_5,54_ = 0.515; *p* = 0.764, NS), suggesting that grooming behavior was not influenced by the experimental conditions. Finally, rearing behavior also showed similar mean values across groups (vehicle: 69.18 ± 7.59; fluoxetine 0.5 mg/kg: 53.67 ± 7.18; fluoxetine 1 mg/kg: 52.90 ± 6.81; HMLH123: 60.51 ± 6.97; I‐1: 61.69 ± 3.45; I‐2: 59.24 ± 6.73), and statistical analysis confirmed no significant differences (*F*_5,54_ = 0.813; *p* = 0.546, NS), indicating that exploratory vertical activity remained unaffected by the treatments.

**Table 1:** Frequency of crossings and duration of grooming and rearing behaviors in the LAT. Values are presented as mean ± standard error for each variable. Statistical analysis was performed using one‐way ANOVA. Abbreviations: I‐1: fluoxetine (0.5 mg/kg) + probiotics HMLH123; I‐2: fluoxetine (1 mg/kg) + probiotics HMLH123.

Treatment	Crossing (*n*)	Grooming (s)	Rearing (s)
Vehicle	50.200 ± 3.829	61.470 ± 11.283	69.179 ± 7.593
Fluoxetine 0.5 mg/kg	48.800 ± 5.831	55.519 ± 8.197	53.672 ± 7.184
Fluoxetine 1 mg/kg	46.400 ± 7.707	58.535 ± 13.292	52.896 ± 6.808
HMLH123	58.000 ± 7.154	57.502 ± 12.768	60.508 ± 6.973
I‐1	66.200 ± 7.786	65.941 ± 8.359	61.686 ± 3.451
I‐2	67.000 ± 10.256	43.238 ± 9.007	59.237 ± 6.726

### 3.3. Forced Swim Test

The analysis of latency to the first immobility revealed significant differences between treatments (*F*_5,54_ = 16.079, *p* < 0.001). Post hoc analysis showed that rats treated with 1 mg/kg fluoxetine, probiotic mixture HMLH123, I‐1, and I‐2 exhibited a significantly longer latency to the first immobility compared to the vehicle group (Figure 2a). Similarly, total immobility time differed significantly between treatments (*F*_5,54_ = 28.768, *p* < 0.001), with the same treatment groups showing a significant reduction in immobility time compared to the vehicle group (Figure 2b). Swimming time also varied significantly between treatments (*F*_5,54_ = 12.832, *p* < 0.001), with rats in the 1 mg/kg fluoxetine, probiotic mixture HMLH123, I‐1, and I‐2 groups displaying a significant increase compared to the vehicle group (Figure 2c). No significant differences were observed in climbing time (*F*_5,54_ = 1.036, *p* = 0.406, NS) (Figure 2d).

Figure 2FST. (a) Latency to first immobility was significantly increased in the Flx 1, Pro, I‐1, and I‐2 groups (*p* < 0.001) compared to Veh, (b) while total immobility time was significantly reduced in these same groups (*p* < 0.001). (c) Swimming behavior was also significantly higher in Flx 1, Pro, I‐1, and I‐2 compared to Veh, (d) whereas climbing showed no significant differences across treatments (*p* < 0.001 vs. Veh, one‐way ANOVA; Student–Newman–Keuls post hoc test). Abbreviations: Veh: vehicle; Flx 0.5: fluoxetine 0.5 mg/kg; Flx 1: fluoxetine 1 mg/kg; Pro: probiotics HMLH123; I‐1: fluoxetine (0.5 mg/kg) + probiotics HMLH123; I‐2: fluoxetine (1 mg/kg) + probiotics HMLH123.

**Figure:**
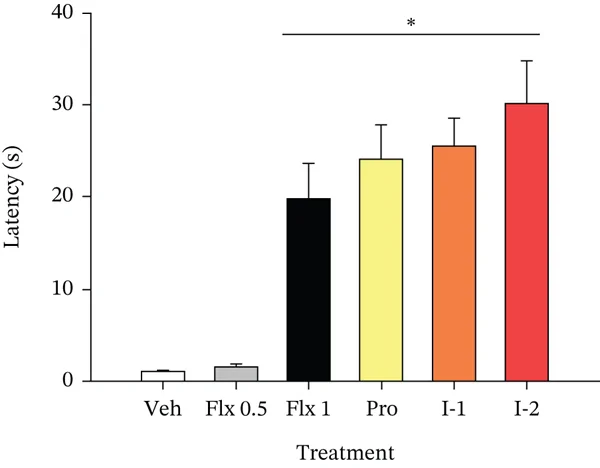
(a)

**Figure:**
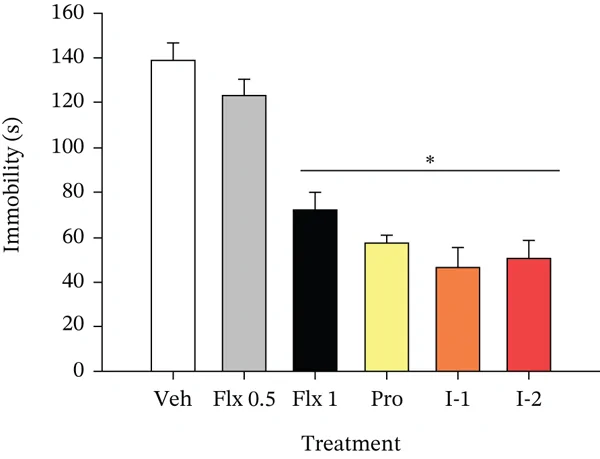
(b)

**Figure:**
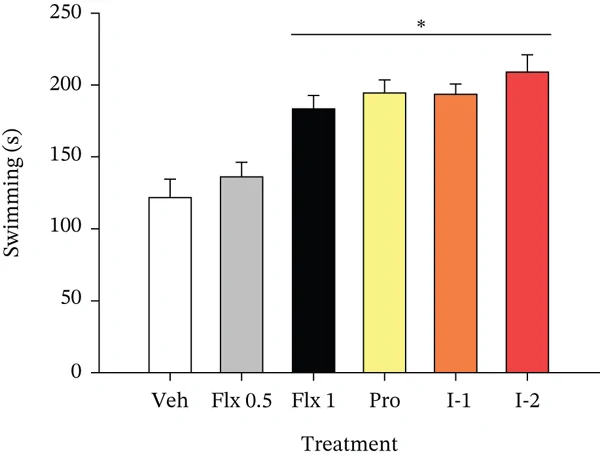
(c)

**Figure:**
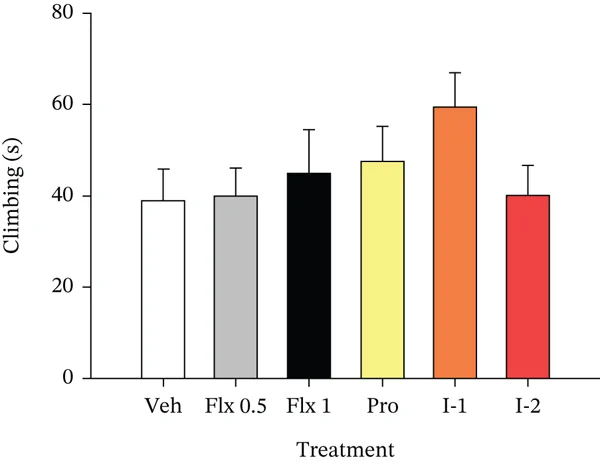
(d)

## 4. Discussion

This study evaluated the effects of a probiotic mixture HMLH123, administered individually and in combination with two different doses of fluoxetine, in animal models of anxiety and depression. The results showed that all treatments increased the time spent in the open arms in the EPM, indicating an anxiety‐reducing effect. In the FST, all treatments reduced immobility time and increased swimming behavior, suggesting a possible modulation of serotonergic neurotransmission without affecting the noradrenergic pathway, as no changes were observed in climbing behavior. These findings reinforce the evidence that probiotics can exert anxiolytic‐ and antidepressant‐like effects comparable to those of selective serotonin reuptake inhibitors (SSRIs) such as fluoxetine [27].

The EPM is widely used to assess substances with potential anxiolytic effects, including hormones, phytochemicals, and pharmaceuticals. In general, an increase in time spent in the open arms is considered an indicator of an anxiolytic effect, as it suggests an anxiety reduction [28]. In this study, the increase in time spent exploring the open arms was observed in the groups treated with fluoxetine, probiotic mixture HMLH123, and their combinations. This increase suggests the anxiolytic‐like effects of these treatments. On the other hand, the effect observed in the FST regarding the possible modulation of serotonergic system by these treatments could explain their anxiolytic properties. In clinical practice, first‐line medications like SSRIs alleviate anxiety symptoms by acting through this mechanism [29]. Our results may suggest that probiotic mixture HMLH123, like fluoxetine, acts on this pathway, highlighting its potential to anxiety‐reducing by a possible modulating serotonin. As both the single treatment and the combined treatments produced similar results, these findings suggest that the probiotic mixture HMLH123 and fluoxetine may share convergent mechanisms in the regulation of anxiety or a possible ceiling effect when both treatments are combined. These findings highlight the need for further neurochemical studies to clear whether probiotic mixture HMLH123 modulates serotonin in key regions of the brain in a similar manner to SSRIs.

The results of the LAT showed that none of the treatments significantly affected the crossings, indicating that the differences observed in the EPM and FST were not due to alterations in general motor activity. This control is crucial to rule out the possibility that the anxiolytic and antidepressant‐like effects observed were the result of nonspecific changes in the animal′s mobility [30].

In FST, prolonged immobility is interpreted as an indicator of behavioral despair, which serves as a preclinical model for depression. The latency to the first episode of immobility reflects the animal′s ability to engage in active behavior before adopting a passive posture [21, 31]. Treatment with antidepressants, including SSRIs, has been shown to reduce the total immobility time and increase latency, which is often associated with increased serotonin availability in the brain [26].

The present results could support these observations, as all treatment groups, including those receiving probiotic mixture HMLH123, exhibited a reduction in immobility and an increase in swimming behavior. These findings suggest that probiotics may produce antidepressant‐like effects like fluoxetine, suggesting a possible modulation of the serotonergic system. The increased swimming behavior observed in all treated groups has been associated in some studies with enhanced serotonergic activity [32].

The results observed with the probiotic mixture HMLH123, characterized by an increase in latency at first immobility, reduction of total immobility time, and increased swimming behavior, are consistent with the findings reported by [13, 33] on *Lactobacillus reuteri*, *Lactobacillus plantarum*, and *Lactobacillus fermentum*. In those studies, interventions appear to positively modulate the serotonergic pathway and reduce immobility behavior in the FST, suggesting a common underlying mechanism. The effect of probiotic mixture HMLH123 on the behavioral parameters associated with depression mirrors what was observed by [34], where *Lactobacillus reuteri* NK33 and *Bifidobacterium adolescentis* NK98 not only alleviated depressive behaviors but also restored neurotransmitter balance.

The increase in latency at first immobility increased by probiotic mixture HMLH123 represents a particularly relevant finding, as it indicates a delay in the manifestation of hopeless behaviors, a central component in depressive disorders. This effect could be related to what was observed by [35] and confirmed in the systematic review by [36], where a correlation is established between the presence of certain beneficial bacteria and the reduction of depressive symptoms. The possible modulation of the serotonergic pathway by the probiotic mixture HMLH123 suggests that this intervention may influence mechanisms associated with mood regulation, potentially resembling some effects of conventional antidepressants. Moreover, this probiotic mixture could contribute to mitigating gut dysbiosis, inflammation, and gut barrier alterations—factors that, according to accumulated evidence, play a crucial role in the pathophysiology of mood disorders. Overall, these findings point to HMLH123 as a promising preclinical approach with potential benefits that warrant further investigation.

The significant increase in swimming behavior observed after administration of the probiotic mixture HMLH123 warrants attention, as it may reflect a possible modulation of serotonergic‐related processes in the FST. This finding aligns with observations by [37] and [38], where probiotics effectively modulated the MGB axis, influencing neurotransmitter metabolism and behaviors related to stress and depression.

An important aspect is that none of the treatments modified climbing behavior in the FST. Previous studies have indicated that increased climbing behavior in this test is often associated with noradrenergic activation, typically observed with tricyclic antidepressants and norepinephrine reuptake inhibitors [31]. The absence of changes in this variable may suggest that both fluoxetine and the probiotic mixture HMLH123 could modulate serotonergic‐related processes, without effects on noradrenergic activity. This observation could be relevant, as treatments influencing norepinephrine are known to sometimes produce side effects such as insomnia, tachycardia, and psychomotor agitation [39].

The lack of a synergistic effect between the probiotic mixture HMLH123 and fluoxetine raises questions about their potential combined use in the treatment of anxiety and depression. Some studies have reported that the combination of microbiota‐modulating agents with conventional antidepressants can enhance the therapeutic response [40]; however, in this study, no additional benefit was observed when both treatments were combined. Although both fluoxetine and HMLH123 alone exert clear antidepressant‐like effects, their combination did not produce a stronger effect than each treatment individually, suggesting a possibly ceiling effect in this behavioral paradigm. Therefore, the present findings suggest that probiotics like HMLH123 may hold preclinical potential to complement conventional antidepressant mechanisms, particularly in contexts where SSRIs show limited efficacy. Future studies are needed to explore this possibility in clinical settings.

Despite the encouraging results, this study presents certain limitations that should be considered when interpreting the findings. One of them is the exclusive use of male rats. Although this approach allowed us to reduce variability and establish a standardized behavioral baseline under controlled hormonal conditions, it limits the generalizability of our findings. Sex differences in anxiety and depression are well documented, potentially due to hormonal fluctuations and differential neurobiological responses to stress [40]. Thus, while excluding females helped minimize the confounding influence of the estrous cycle in this initial screening, future research should incorporate both sexes to explore the modulatory role of ovarian hormones and to determine whether HMLH123 exerts sex‐dependent effects. An important limitation of this study is that behavioral data alone cannot establish the specific mechanisms underlying the observed antidepressant‐like effects of HMLH123. While the increased swimming behavior in the FST is consistent with serotonergic modulation, multiple alternative or complementary pathways could produce identical behavioral phenotypes, including peripheral serotonin synthesis, anti‐inflammatory effects, HPA axis regulation, gut‐derived metabolite production (e.g., SCFAs), neurotrophic factor upregulation, or synergistic combinations thereof. The MGB axis involves complex bidirectional communication through neural, endocrine, immune, and metabolic channels that cannot be differentiated through behavioral assessment alone. Therefore, our findings demonstrate therapeutic‐like behavioral outcomes but do not establish mechanistic causality. Future research incorporating neurochemical analyses, microbiota characterization, metabolite profiling, inflammatory marker assessment, and pharmacological antagonism studies will be essential to determine which pathway(s) mediate these effects and to elucidate the precise mechanisms of action.

Another limitation of this study is the absence of fecal microbiota analysis, such as 16S rRNA sequencing, which would have allowed a direct assessment of gut microbial changes associated with HMLH123 administration. In addition, environmental factors such as diet and housing conditions could have influenced microbiota composition and, consequently, the observed effects. Considering the relevance of the gut–brain axis in modulating emotional behaviors, characterizing microbial community dynamics—including shifts in diversity, the relative abundance of specific taxa, and their potential correlations with behavioral outcomes—would provide valuable mechanistic insights into the observed effects. This study was intentionally limited to behavioral assessments to establish the anxiolytic‐ and antidepressant‐like profile of HMLH123 as an initial step. Nonetheless, future research should integrate comprehensive microbiota analyses to elucidate the microbial mechanisms underlying these behavioral effects. Finally, it is important to acknowledge that although the experimental design and statistical analysis are appropriate for an independent‐groups framework, the results can only suggest the presence of a potential synergistic effect between fluoxetine and HMLH123. However, the absence of a factorial design capable of formally evaluating the interaction between both compounds prevents us from demonstrating this synergistic effect conclusively. Therefore, the findings should be interpreted with caution.

## 5. Conclusions

The results obtained suggest that the probiotic mixture HMLH123 may exert anxiolytic and antidepressant‐like effects like those of fluoxetine by potentially modulating serotonergic activity without affecting the noradrenergic system. However, since no synergistic effect was observed between the treatments, probiotics could be considered as a potential option in cases where patients do not adequately respond to first‐line treatments such as SSRIs. Nevertheless, further research is needed to fully understand how probiotics could modulate serotonergic neurotransmission and to assess their clinical viability in this context.

## Author Contributions

Gilberto Uriel Rosas‐Sánchez, León Jesús Germán‐Ponciano, and Cesar Soria‐Fregozo were responsible for the design and conceptualization of the study. Angélica Yanet Nápoles‐Medina and Blanca Rosa Aguilar‐Uscanga prepared the probiotics used in the study. Gilberto Uriel Rosas‐Sánchez and Cesar Soria‐Fregozo conducted the experiments. Data analysis was performed by Gilberto Uriel Rosas‐Sánchez, León Jesús Germán‐Ponciano, and Cesar Soria‐Fregozo. Gilberto Uriel Rosas‐Sánchez, León Jesús Germán‐Ponciano, Cesar Soria‐Fregozo, and Mario Eduardo Flores‐Soto contributed to data interpretation and manuscript drafting.

## Funding

The study is supported by Secretaría de Ciencia, Humanidades, Tecnología e Innovación (714866 and 1099710).

## Disclosure

All authors reviewed and approved the final version of the manuscript.

## Conflicts of Interest

The authors declare no conflicts of interest.

## Data Availability

Herein, data supporting the findings and any related information is available from the corresponding author upon reasonable request.
